# Eruptive collagenoma in a juvenile patient with Down syndrome^☆^

**DOI:** 10.1016/j.abd.2020.10.026

**Published:** 2022-07-09

**Authors:** Yasunobu Kato, Toshiyuki Yamamoto

**Affiliations:** Department of Dermatology, Fukushima Medical University, Fukushima, Japan

Dear Editor,

Eruptive collagenoma is a rare connective tissue nevus, which presents with asymptomatic, multiple, discrete, firm, slightly elevated, skin-colored, or reddish papules on the trunk and extremities. We describe herein a rare case of eruptive collagenoma on the back of a juvenile patient with Down syndrome.

A 14-year-old boy with Down syndrome and a previous history of anorectal anomaly visited our Dermatology Department, complaining of asymptomatic eruption on his back. He had no family history of connective tissue nevi. Physical examination showed several red-brown or whitish papules on his back (Fig. 1). There were no eruptions in other locations. One of the papules was removed under local anesthesia. A biopsy specimen revealed relatively well-circumscribed areas with increased collagenous fibers in the upper to mid-dermis (Fig. 2A). Lack of elastic fibers as compared with surrounding dermis was observed in Elastica Masson (Fig. 2B), Elastica van Gieson and Weigert staining. A diagnosis of collagenoma was made. No specific treatment was given.

**Figure 1:**
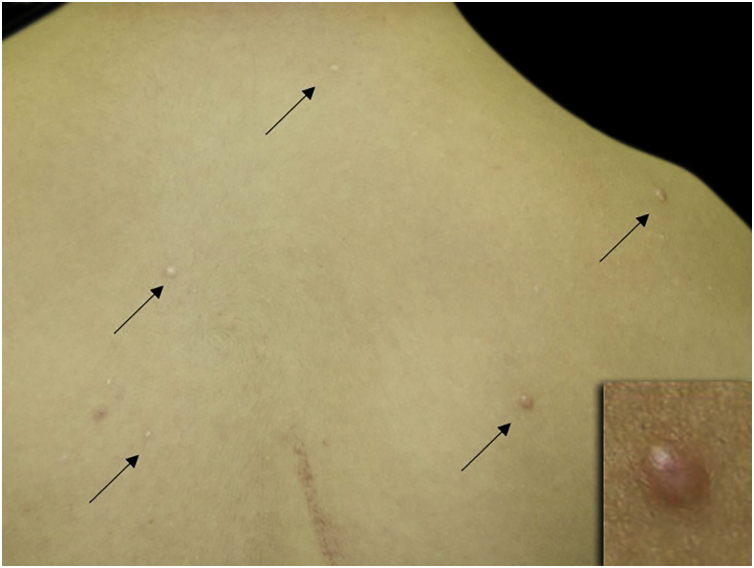
Small oval, slightly reddish or skin-colored papules scattered on the back (arrow).

**Figure 2:**
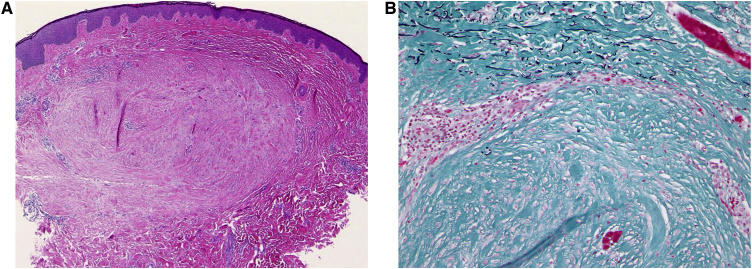
(A), Histological features showing thickened collagen bundles in the dermis (Hematoxylin & eosin staining). (B), Elastica Masson staining revealed that elastic fibers were observed in the surrounding. (Original magnification: A; ×40, B; ×200).

Several cutaneous manifestations are associated with Down syndrome, including atopic eczema, seborrheic eczema, alopecia areata, vitiligo, psoriasis, tinea, syringoma, and milia-like calcinosis cutis.^1^ By contrast, connective tissue disorders such as collagenoma, connective tissue nevi, anetoderma, and elastosis perforans serpiginosa, are rare. Eruptive or solitary collagenoma is rarely seen in patients with Down syndrome.^2^, ^3^, ^4^ Among the previously reported 5 patients and our patient, 2 were male, and 3 were pediatric patients; and age ranged between 7 and 47 years old. The affected sites were the neck, chest, back, abdomen, buttock, groin, sacrococcygeal region, thighs, hands, and arms. Four patients developed multiple lesions, while 2 patients developed solitary lesions. The etiology of collagenoma in Down syndrome remains unknown; however, premature aging due to impaired DNA repair or altered free radical metabolism may be involved.^1^ The genetic locus for superoxide dismutase, a key enzyme in free radical metabolism is located in chromosome 21. Moreover, in the skin of fetuses with trisomy 21, overexpression of COL6A1, and irregular arrangement of type VI collagen were observed.^5^

Differential diagnosis mainly includes papular elastorrhexis and nevus anelasticus. Papular elastorrhexis occurs during the first or second decades of life and presents with asymptomatic, small, non-follicular, creamy-white papules on the chest, abdomen, and back. Histopathology shows fragmented elastic fibers. Nevus anelasticus is an acquired nevus characterized by perifollicular papules and histopathologically, fragmentation or loss of elastic fibers. Collagenomas can be found in association with hereditable conditions such as Multiple Endocrine Neoplasia type 1 (MEN1). The present case did not have either a family history of MEN1 or tumors of parathyroid glands, endocrine pancreas and anterior pituitary.

In conclusion, we report a rare case of eruptive collagenoma in an adolescent patient with Down syndrome. Further studies are necessary to elucidate the mechanism of collagenoma in association with Down syndrome.

## Financial support

None declared.

## Authors’ contributions

Yasunobu Kato: Played a role in conception, design, analysis, and approval; read and approved the final version of the manuscript.

Toshiyuki Yamamoto: Played a role in conception, analysis, writing, and approval; read and approved the final version of the manuscript.

## Conflicts of interest

None declared.

## References

[1] Madan V., Williams J., Lear J.T. (2006). Dermatological manifestations of Down’s syndrome. Clin Exp Dermatol.

[2] Choi S.Y., Park S. (2018). Collagenoma in a patient with Down syndrome: a case report and review of the literature. Am J Dermatopathol.

[3] Smith J.B., Hogan D.J., Glass L.F., Fenske N.A. (1995). Multiple collagenomas in a patient with Down syndrome. J Am Acad Dermatol.

[4] Togawa Y., Nohira G., Shinkai H., Utani A. (2003). Collagenoma in Down syndrome. Br J Dermatol.

[5] Kaisenberg C.S., Brand-Saberi B., Christ B., Vallian S., Farzaneh F., Nicolaides K.H. (1998). Collagen type VI gene expression in the skin of trisomy 21 fetuses. Obstet Gynecol.

